# Emergency department screening and interventions for substance use disorders

**DOI:** 10.1186/s13722-018-0117-1

**Published:** 2018-08-06

**Authors:** Kathryn Hawk, Gail D’Onofrio

**Affiliations:** Department of Emergency Medicine, Yale University, 464 Congress Ave, Suite 260, New Haven, CT 06519 USA

## Abstract

The emergency department (ED) has long been recognized as providing critical access to the health care system for many, yet only in the past few decades has the ED visit been recognized as an opportunity to identify and link patients to care for substance use disorders (SUDs). This review explores the evidence for ED-based screening, psychosocial and pharmacological interventions, and linkage to treatment for the spectrum of SUDs including high risk alcohol use and alcohol, opioid, tobacco and other SUDs. Despite knowledge gaps, methodological challenges and some inconsistency across interventions studied, opportunities for EDs to improve the care of patients across the spectrum of SUDs are robust.

## Background

The emergency department (ED) has long been recognized as a front door to the hospital and as providing critical access to the health care system for many, yet only in the past few decades has the ED visit been recognized as an opportunity to identify and link patients to care for substance use disorders [1–3]. Increasingly, the practice of actively screening, initiating psychosocial and pharmacological interventions, and linking patients with substance use disorders (SUD) to effective treatment has become more common, but for a variety of reasons, many EDs have not embraced the critical part they can play in this process [4].

Individuals with SUDs regularly access emergency care, with nearly half of all ED visits in the US categorized as relating to substance use disorders [5]. EDs disproportionately provide medical care for individuals with SUDs, thus offering access to the 20.1 million Americans aged 12 and older who meet criteria for a SUD [6]. The National Survey on Drug Use and Health (NSDUH) data from 2016, show that of the 19.9 million adults who needed treatment for a substance use disorder, only 2.1 million, or 10.8%, received addiction treatment within the past 12 months [7]. A Healthcare Cost and Utilization Project (HCUP) analysis on trends from 2006 to 2013 in the rate of ED visits involving substance use disorders, found a 37% increase, from 1838 to 2519, ED visits per 100,000 people ≥ 15 years of age [8].

Costs associated with SUDs, including lost productivity, healthcare costs and crime, are staggering and were reported as exceeding 400 billion dollars per year in the 2016 US Surgeon General’s Report, Facing Addiction in America [9]. This burden could potentially be reduced by closing the treatment gap, thus reducing increased costs from medical complications of SUDs, as well as higher ED admission rates, more frequent unintentional injuries, motor vehicle collisions, interpersonal violence, HIV and intentional or accidental overdose [10–13].

An ED visit for an acute change in health, whether from SUD related injury, pneumonia, soft tissue infection or overdose, provides an opportunity for physicians to actively engage patients in discussion and reflection, to help them to make the connection between substance use and their acute medical condition, which may help provide motivation for behavior change. Sometimes this connection is evident to patients, but often it is not, and concepts derived from motivational interviewing (MI) have been adapted to brief interventions used in ED settings to engage individuals in the process of making positive behavior changes through a 4 step process of engaging, focusing, evoking and planning [14, 15]. These interventions help guide the participant towards resolution of ambivalence and internal inconsistencies in an empathetic setting, and patients are assisted in making the connection between substance use and outcomes. Brief interventions are thus motivational interview-based conversations, that are empathetic, non-judgmental, patient autonomy centered, and often include MI based principals of open-ended questions, affirmations, reflective listening and summaries [16, 17]. There is a reasonably large body of literature on the effectiveness of brief intervention in the ED setting, with mixed and sometimes directly conflicting results, complicated by problematic methodology, fidelity to the intervention, concerns about assessment reactivity, variation in intervention dose, and intervention application across heterogeneous populations using a variety of different outcomes [17–25].

## SUD screening recommendations and tools

The U.S. Preventative Services Task Force recommends screening in primary care settings for adults 18 years and older for alcohol misuse and advises brief behavioral counseling interventions for those engaged in risky or hazardous drinking to reduce alcohol misuse; no recommendations are made about ED care [26]. The American College of Emergency Physicians (ACEP) published in 2005 and then reaffirmed in 2017 a policy statement on alcohol screening in the ED: “ACEP believes emergency medical professionals are positioned and qualified to mitigate the consequences of alcohol abuse through screening programs, brief intervention, and referral to treatment” [27]. There is no policy recommendation on broad based brief intervention and referral to treatment for the treatment of other drugs of abuse in the Emergency Department.

The development of abbreviated validated tools to screen for alcohol enhances the ability for EDs to implement screening for alcohol misuse and alcohol use disorders. These tools include the 3 question AUDIT-C [28, 29], the CAGE [30, 31] (cut down, annoyed, guilty, eye-opener) questionnaire and the National Institute on Alcohol Abuse and Alcoholism (NIAAA) single question screen: ““How many times in the past year have you had 5 [for men] or 4 [for women and all adults older than 65 years] or more drinks in a day?”, [32] and have been recommended by organizations and used in multiple trials” [16, 26, 33].

**Fig. 1:**
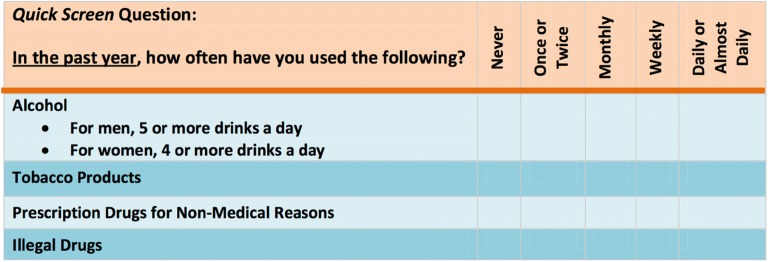
NIDA Quick Screen for substance use disorders. If the patient says **“No”** for all drugs in the Quick Screen, reinforce abstinence. **Screening is complete.** If patient says **“Yes”** to **one or more days of heavy drinking**, note that *patient is an at*-*risk drinker*. If patient says **“Yes”** to **use of tobacco**: *Any* current tobacco use places a patient at risk. If the patient says **“Yes”** to **use of illegal drugs or prescription drugs for non-medical reasons**, proceed to **Question 1** of the NIDA-Modified ASSIST. Adapted from NIDA Screening for Drug Use in General Medical Settings Resource Guide [67, 68]

Validated screening tools for substance use disorders including the 10 item Drug Abuse Screening Test (DAST) [34, 35] exist, but the abbreviated NIDA Quick Screen Single drug use question: “How many times in the past year have you used an illegal drug or used a prescription medication for nonmedical reasons”, may be more appropriate in ED settings. This single screening question was found to be 100% sensitive and 73.5% specific for the detection of a drug use disorder in a primary care setting [36] (Fig. 1).

## ED interventions for alcohol

Over the past several decades, a large body of literature has emerged relating to screening for alcohol and ED based interventions across the spectrum of patients with alcohol use and alcohol use disorders including harmful, hazardous, at-risk and dependent drinkers. Interventions focused on at-risk, harmful, hazardous drinkers have largely focused on reducing frequency or quantity of alcohol use, as well as injury prevention (i.e. alcohol-related injury, reduced drinking and driving, increasing seat belt use, etc.), while interventions focused on dependent drinkers or those meeting criteria for moderate or several alcohol use disorder focus on enhancing motivation to enter treatment [17, 18, 20, 37, 38]. Importantly, one observational study found that patients who received a direct referral, including transfer directly from the ED to a specialized treatment facility, were 30 times more likely to enroll in treatment than those with an indirect referral who are discharged home from the ED [38] (Table 1).

**Table 1:** Spectrum of alcohol use and alcohol use disorders [39, 40]

	Definition
At-risk drinking	Pattern of alcohol consumption that exceeds NIAAA recommendations occasionally to frequently
Harmful drinking	Pattern of alcohol use that causes mental or physical damage
Hazardous drinking	Pattern of alcohol consumption that increases one’s risk of harm
Alcohol use disorder	Meets ≥ 2 DSM-V criteria for alcohol use disorder

Although results are conflicting, in part due to heterogeneity across study populations and outcomes, the literature overall supports the use of motivational interview-based interventions for alcohol use in the emergency department. In 1997, a single site observational study of a brief intervention-based program found a significant reduction in frequency of alcohol use, drinks per day and frequency of > 5 drinks in one sitting in harmful and hazardous drinkers [41], while a 14 site study of screening and brief intervention for high risk alcohol consumers found a significant reduction in weekly drink consumption at 3 month follow-up in the group receiving a MI based intervention called the Brief Negotiated Interview (BNI) [2, 21]. A 2007 systematic review and meta-analysis examining the effect of interventions for alcohol problems in the ED identified 13 studies targeting reduction of alcohol consumption and related harm in non-alcohol dependent ED patients, and found no effect on the quantity/frequency or drinking or the frequency of heavy drinking at 12 months, inconclusive effects on the frequency of heavy drinking at 3 months, but a 41% reduction in the odds of sustaining an alcohol related injury in the 6 and 12 months following the index ED intervention [18].

A 2008 study of harmful and hazardous drinkers did not detect a difference between patients receiving a BNI and scripted discharge instructions [42], although a second study of harmful and hazardous drinkers, that included universal screening and interactive voice response methodology to reduce assessment reactivity found reduction in mean past 7-day alcohol use and past 28 day binge episodes at 6 and 12 month follow-up in the BNI group and BNI group with 30 day booster over standard care [17]. A 2017 systematic review of ED studies using brief intervention or motivational interview based intervention to reduce alcohol consumption identified 25 randomized controlled trials; 13 studies showed decreased alcohol consumption at primary outcome; 17 studies failed to demonstrate intervention effect for primary outcome of alcohol consumption, 11 of which found significant results for either a specific subgroup or a secondary outcome [37]. Overall, authors conclude that there is moderate quality evidence for the targeted use of brief interventions that showed a small reduction in alcohol use in low or moderate drinkers and a reduction in the consequences of use such as injury [37].

Despite some inconsistencies in effectiveness studies of ED brief intervention with referral to treatment with a focus on reducing alcohol use and injury, the literature demonstrates an overall trend toward cost-effectiveness [24, 43, 44]. A 2005 cost–benefit analysis of injured ED and hospitalized patients advised routine implementation of screening and brief intervention of all trauma patients after finding that 27% of all injured adult patients in the study were candidates for a brief intervention for alcohol, and factoring in health care systems level costs estimated $3.81 saved for every $1 spent on screening and intervention [44]. Additionally, an evaluation of working-age, disabled Medicaid patients in Washington State who received screening and a brief intervention that included a referral to SUD treatment if indicated found a reduction of $366 in Medicaid costs per member per month after propensity matching [43]. A more recent study calculated healthcare costs (total health care costs, 30-day ED visits, 1-year ED visits, inpatient claims, and behavioral health claims) at multiple EDs matched on location and time to a single ED that offered SBIRT to enhance SUD treatment and found a 21% reduction in health care costs in the cohort who received SBIRT, which translated to $2100 per patient receiving SBIRT per year [24].

## ED interventions for non-medical opioid and illicit drug use

Early studies for MI based interventions on illicit drug use were promising, though evidence supporting the effectiveness of brief intervention in the ED setting to improve illicit drug related outcomes is limited. In general, there is a paucity of evidence supporting MI based interventions for illicit drug use in general, but compelling evidence exists specifically for ED-interventions specifically targeted for opioid use disorder (OUD) [3, 45]. A 2005 study demonstrated biochemical confirmation of increased cocaine and heroin abstinence at 6 months in the brief intervention arm of a RCT conducted in an urgent care, women’s clinic and homeless clinic setting [46]. An analysis of a large, multi-site study using brief intervention in ED and primary care settings of patients with illicit drug use at baseline reported a 67% reduction in illict drug use in those receiving brief intervention [47]. More recently, a large multisite trial prospectively evaluated the effect of randomization to brief intervention with booster versus screening, assessment and referral versus minimal screening only found no difference either in self-reported or biochemical confirmed drug use at 3, 6 or 12 months [22]. Important factors confounding the interpretation of this study include the, heterogeneity of drug type and severity use in the sample, and limited fidelity to the planned MI intervention with only 57% of participants in the MI treatment arm receiving the first booster and 39% of participants receiving the 2nd booster [22]. A large, single site study found that brief intervention for ED patients with drug or alcohol use disorders did not improve attendance at post-ED intervention over a case management intervention [48].

Two recent studies have demonstrated improved opioid-related outcomes after ED intervention for OUD. A pilot randomized clinical trial of 204 ED patients reporting non-medical prescription opioid use within the past 3 months found reductions in overdose risk behaviors and non-medical opioid use in the MI-intervention group in comparison to enhanced usual care [45]. A 2015 randomized clinical trial of ED patients with opioid dependence found a significant increase in treatment engagement at 30 days for patients randomized to the brief intervention, buprenorphine-induction and primary care follow up group (78%) in comparison to brief intervention and facilitated referral (45%) and referral to treatment (38%) [3]. Brief intervention with ED-initiated buprenorphine and primary care follow-up was also associated with decreased self-reported past 7 day opioid use at 30 day follow up [3], and cost-effectiveness using a healthcare systems perspective across all willingness-to-pay thresholds at 30 days [49].

This study has influenced a rapidly evolving clinical practice as many emergency departments are increasingly treating opioid withdrawal with buprenorphine and actively linking ED patient with opioid use disorder to care by starting buprenorphine in the ED and referring to treatment [50, 51].

Emerging evidence on the effectiveness of overdose prevention education and community naloxone distributions to individuals likely to witness or experience an overdose laid the foundation for the integration of overdose prevention and naloxone distribution into emergency departments who provide care to particularly high-risk patient populations, including those with ED visits for non-fatal overdose [52–57]. Descriptive studies about implementation of overdose prevention and naloxone distribution to ED patients at risk for opioid overdose have been reported, indicating feasibility and acceptability of overdose prevention and naloxone distribution from the ED to patients at risk of opioid overdose, though systematic prospective studies of ED overdose prevention and naloxone distribution have not been published [58, 59]. These programs are supported by an April, 2018 advisory from Jerome Adams, the 20th US Surgeon General that broadly supports clinicians to prescribe or dispense naloxone to individuals at risk of opioid overdose and their friends and family and “increase the awareness, possession and use of naloxone among at risk populations and broader communities” [60].

Some EDs are integrating the use of a peer navigator or recovery coach into post-opioid overdose ED care, though investigations on effectiveness have not been published to date [61].

## ED interventions for tobacco

A meta-analysis of the literature through 2010 evaluating the effect of ED-initiated tobacco control interventions including preventative health services such as brief interventions and treatment referrals for smoking cessation identified 7 trials of weak to moderate quality and found the strongest effect of point prevalence of tobacco abstinence at 1 month (*RR *=1.47 (3 studies) (95% CI 1.06–2.06)), with a trend towards increased episodically measured tobacco abstinence up to 12 months (*RR *=1.33 (7 studies) (95% CI 0.96–1.83), *P* = 0.08) [62]. A randomized controlled trial published in 2011 found no difference between usual care who received screening and a brochure and enhanced care arm, who received a motivational interview, nicotine patches, and a booster call, although authors attributed the negative outcome to higher than expected quit rates in the usual care group, and hypothesized that low intensity screening and referral may have triggered some smokers to quit [63]. Interestingly, factors associated with quitting included any tobacco related ICD-9 ED diagnosis at index ED visit or subject belief that ED visit was tobacco releated [63]. A follow-up study of 778 low-income emergency department patients found that patients in the intervention group, who received a brief intervention, 6 weeks of nicotine replacement therapy, referral to a quitline had significantly higher rates of biochemically confirmed abstinence at 3 months (12.2%) compared to those who only received a brochure (4.9%). An updated meta-analysis containing 11 studies (10 published studies, 1 abstract) found a significant effect on the 1-year combined point prevalence of (RR of 1.40 (95% CI 1.06–1.86) (*P* = 0.02) [64]. Together, these results suggest that the ED provides a teachable moment about the relationship between a patients symptoms and his or her tobacco use, which can lead to sustained changes in tobacco-related behaviors.

## Conclusion

Although opportunities exist to identify and refine effective ED care of patients with SUDs, the importance that the ED can have in improving outcomes for patients with SUDs is clear. Ample opportunities exist for emergency providers to improve care by screening, initiating treatment, either psychosocial or pharmacotherapies, and directly linking patients to ongoing treatment. Barriers to effective ED management of SUDs include competing priorities, inadequate training in addiction medicine, and stigma, some of which can be overcome by increasing the quantity and quality of addiction medicine training in the medical, nursing and allied health sciences training and post-graduate education, and by prioritization of enhanced care of the ED patient with SUDs through national and local reimbursement and quality mechanisms [65, 66].
