# Correction to: Emergency department screening and interventions for substance use disorders

**DOI:** 10.1186/s13722-019-0155-3

**Published:** 2019-07-16

**Authors:** Kathryn Hawk, Gail D’Onofrio

**Affiliations:** Department of Emergency Medicine, Yale University, 464 Congress Ave, Suite 260, New Haven, CT 06519 USA

## Correction to: ﻿Addict Sci Clin Pract (2018) 13:18 10.1186/s13722-018-0117-1

In the version of this article that was originally published [1], some information in the “Background” section was erroneously omitted. This has now been corrected in this Correction note.

## Background

Individuals with SUDs regularly access emergency care, with nearly half of all 4.9 million drug-related ED visits in 2010 categorized as relating to substance use disorders [5].
